# Laboratory-Acquired Vaccinia Virus Infection in a Recently Immunized Person — Massachusetts, 2013

**Published:** 2015-05-01

**Authors:** Christopher H. Hsu, Julien Farland, Thomas Winters, Julia Gunn, Donna Caron, Jennifer Evans, Lynda Osadebe, Leon Bethune, Andrea M. McCollum, Nishi Patel, Kimberly Wilkins, Whitni Davidson, Brett Petersen, M. Anita Barry

**Affiliations:** 1 Epidemic Intelligence Service, CDC; 2 Division of High-Consequence Pathogens and Pathology, CDC; 3 Boston Public Health Commission; 4 Occupational Environmental Health Network

On November 26, 2013, the CDC poxvirus laboratory was notified by the Boston Public Health Commission (BPHC) of an inadvertent inoculation of a recently vaccinated (ACAM2000 smallpox vaccine) laboratory worker with wild type vaccinia virus (VACV) Western Reserve. A joint investigation by CDC and BPHC confirmed orthopoxvirus infection in the worker, who had reported a needle stick in his thumb while inoculating a mouse with VACV. He experienced a non-tender, red rash on his arm, diagnosed at a local emergency department as cellulitis. He subsequently developed a necrotic lesion on his thumb, diagnosed as VACV infection. Three weeks after the injury, the thumb lesion was surgically debrided and at 2 months post-injury, the skin lesion had resolved. The investigation confirmed that the infection was the first reported VACV infection in the United States in a laboratory worker vaccinated according to the Advisory Committee on Immunization Practices (ACIP) recommendations. The incident prompted the academic institution to outline biosafety measures for working with biologic agents, such as biosafety training of laboratory personnel, vaccination (if appropriate), and steps in incident reporting. Though vaccination has been shown to be an effective measure in protecting personnel in the laboratory setting, this case report underscores the importance of proper safety measures and incident reporting (1,2).

## Case Report

**FIGURE 1:**
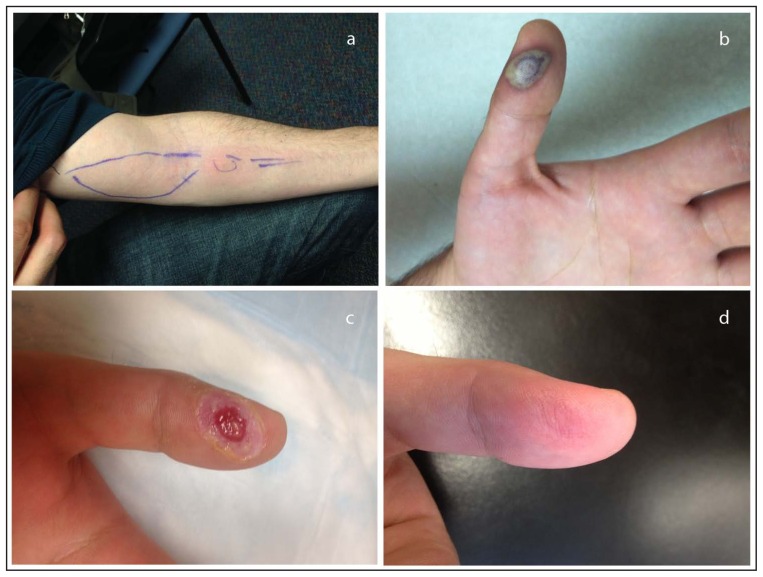
Progression* of vaccinia virus (VACV) infection in VACV-immunized laboratory worker inadvertently inoculated with VACV — Massachusetts, 2013 * a) erythema along left bicep 6 days post-inoculation, b) lesion on left thumb 9 days post-inoculation, c) lesion on left thumb after surgical debridement 23 days post-inoculation, d) left thumb exhibiting complete resolution of infection >3 weeks after surgical debridement.

On November 23, 2013, a man aged 27 years who was a laboratory worker at an academic institution went to a local emergency department with a non-tender, erythematous rash on the skin over his left biceps and extending to the antecubital fossa (Figure 1a). He reported a needle stick in his left thumb had occurred on November 17 while he was inoculating a mouse by scarification with VACV. He had no fever, chills, or other systemic or neurologic symptoms. An ultrasound of his left thumb revealed a small collection of fluid at the puncture site. No culture was performed. Cellulitis was diagnosed in the patient, and he was admitted to the hospital and given cefazolin intravenously, 1 g every 6 hours for 18 hours. He was discharged on November 24 with a prescription for cephalexin, 500 mg orally four times a day for 10 days. A dressing was placed over the wound, and he was instructed to change the dressing three times a day and dispose of the contents in a biohazard container provided by the hospital. He was also instructed to report the next day to the occupational health clinic at the institution where he worked.

On November 25, the patient went to the institution’s occupational health clinic with a necrotic lesion on the volar surface of the left thumb and erythema over the left biceps extending to the volar forearm. A necrotic VACV infection was diagnosed, and the patient was advised to continue cephalexin. As required by BPHC research laboratory regulations, occupational health notified BPHC, which notified the Massachusetts Department of Public Health and CDC. BPHC initiated an investigation and reinforced infection control measures, including instruction on keeping the wound covered and proper disposal of dressings.

An evaluation on November 26 revealed that the necrotic lesion on the thumb persisted (Figure 1b), but erythema of the arm was less pronounced. A blood specimen was sent to the CDC for serological and molecular testing. By November 27, the lesion appeared stable and the erythema had resolved.

**FIGURE 2:**
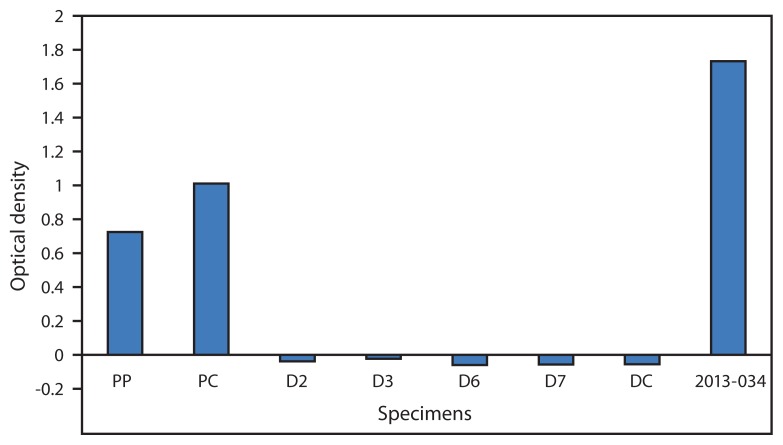
Results of serologic testing for vaccinia virus (VACV) in a VACV-immunized laboratory worker inadvertently inoculated with VACV — Massachusetts, 2013 **Abbreviations:** PP = immunoglobulin (Ig) G positive pool; PC = IgG positive control; D2, D3, D6, D7, DC = negative controls; 2013-034 = laboratory worker’s specimen.

On December 10, 23 days after the injury, the lesion was surgically debrided (Figure 1c) and a specimen was submitted for diagnostic testing at Hinton State Laboratory Institute and CDC. Orthopoxvirus infection was confirmed at both laboratories using polymerase chain reaction (3). VACV was isolated using tissue culture at CDC (4). Serology completed by CDC revealed high levels of orthopoxvirus immunoglobulin G (Figure 2) (5). By January 9, 2014, the skin lesion had resolved (Figure 1d), and the patient was asymptomatic.

## Exposure History and Laboratory Safety Evaluation

Investigation by BPHC found that on November 17, 2013, the patient sustained a needle-stick injury on his left thumb while recapping a 25-gauge needle. The needle had been used to scarify mice with non-recombinant wild type VACV Western Reserve type 1354. The experiment involved applying 10 *μ*L of 10^5^ plaque-forming units/*μ*L of trypsinized virus stock on mouse skin and using an empty needle to inoculate by scarification.

Mice were anesthetized during the procedure, and the experiment was performed in a Class II biosafety cabinet. The patient reported that as he performed the scarification procedure on the anesthetized mouse, a mouse in an adjacent cage distracted him. When he attempted to recap the needle, it penetrated two layers of gloves and punctured the volar surface of his left thumb. He immediately sprayed his gloves with a chlorine dioxide-based sterilant, removed the gloves, degowned, and washed his hand with water and soap for approximately 10 minutes, expressing blood from the injury as he washed his hand. The gloves were examined immediately after the needle-stick. He noticed a visible hole and small amount of blood. An incident report was filed with the project’s principal investigator on November 17, the day of the needle-stick injury. The principal investigator subsequently contacted an infectious disease physician, who advised that the patient should go immediately to a hospital emergency department if there were signs of infection.

BPHC staff visited the institution on November 26, 2013, as part of the investigation. The biologic safety officer, laboratory manager, principal investigator, occupational health nurse, and patient were present. BPHC toured the animal facility and the research laboratory noting that both areas were well maintained, with proper biosafety signage, certified biosafety cabinets, disinfectants, and waste containers. The laboratory protocols and the VACV vaccination recommendations for staff were also reviewed by BPHC, which identified the practice of recapping needles as a lapse from standard laboratory procedure.

The patient had been working in the laboratory since January 2013 and working with VACV since March 2013. In January 2013, he completed both New Employee Safety Training and Animal Use Orientation, which included animal biosafety. On March 22, he had received individualized, specific VACV training, including work practices and procedures related to working with VACV. Potential routes of exposure, vaccination, monitoring of vaccination response, emergency procedures, and incident reporting were covered in this training. The patient had also met with an animal care supervisor to review the established animal care procedures for the laboratory.

As of January 2014, the laboratory affirmed its intent to use safety syringes and needles in future experiments, and the academic institution outlined measures to be taken to ensure safe use of biologic agents, which included discouraging recapping of needles, reviewing biosafety-level 2 animal inoculation procedures by animal care staff, and providing information pertaining to the availability of safety needles for use in research. The required training for all research principal investigators was revised by the institution to emphasize their responsibilities in incident/injury reporting for staff working with biologic materials under the Institutional Biosafety Committee’s purview.

The patient had been vaccinated with the ACAM2000 smallpox vaccine on January 28, 2013 (confirmed by medical record review and physician recall). A new vial of vaccine had been reconstituted that day, just before use. On February 5, 2013, 9 days after vaccination, the patient was evaluated at the occupational health facility where he had received his vaccination. At that time a 0.5-cm white lesion was present at the center of the vaccination site (left deltoid). Wound edges were pink but intact. Scant yellow/green drainage was observed on the dressing. At follow-up a week later, a 0.5-cm brown dry eschar was present at the center of the wound. These findings were consistent with a major cutaneous reaction, or “take,” suggesting a successful response to vaccination. The vaccine from this vial was also administered to two other recipients with no reported vaccine failures. Previously, five other researchers in the laboratory had also been offered and accepted vaccination.

### Discussion

The ACIP recommends smallpox vaccination for laboratory personnel who directly handle cultures or animals contaminated or infected with non-highly attenuated VACV (1). Persons working with non-highly attenuated VACV (e.g., Western Reserve) or non-variola orthopoxviruses are recommended to be revaccinated every 10 years; persons working with more virulent non-variola orthopoxviruses such as monkeypox can consider revaccination every 3 years to ensure adequate protection (1). Laboratory-acquired VACV infections have been reported previously (2); however, this is the first report of laboratory-acquired VACV infection in a recently vaccinated laboratory worker. Two other cases of laboratory-acquired VACV among vaccinated persons have been reported, but in one case, the person was vaccinated >10 years before exposure, thus not conforming to ACIP recommendations, and the other did not exhibit a vaccine take at the time of vaccination, which was 6 years before exposure (6). Vaccination with VACV is administered by scarification of the skin which causes characteristic focal lesions that are indicative of successful vaccination, otherwise known as a major cutaneous reaction, or take (7,8). Cutaneous reactions at the inoculation area can include a papule, vesicle, ulcer, or crusted lesion surrounded by induration (8).

The patient’s elevated levels of immunoglobulin G indicate prior exposure by vaccination or infection. However, the level of antibody that protects against VACV infection is unknown and antibody level might not be indicative of protective, neutralizing antibodies against infectivity (9). The viral load caused by the patient’s needle stick and the significance it played in clinical symptomology are also unknown. Knowing the viral load in the patient might have helped explain why the patient experienced symptoms despite having been vaccinated. In addition, vaccination might not offer full immunity but might lessen clinical severity as evidenced by amelioration or absence of takes in re-vaccinees (9). Administration of VACV within a few days of exposure to smallpox virus has been shown to reduce symptoms of disease (1), so it remains a possibility that this patient’s infection was reduced in severity because of preexisting immunity. This underscores the importance of smallpox vaccination among laboratory workers who use VACV in research settings, which is recommended by ACIP to prevent or minimize the effects of unintentional orthopoxvirus infection in a laboratory (10). Finally, establishing and reinforcing safe laboratory practices such as proper handling of contaminated needles and use of personal protective equipment is important in reducing the risk of injury and infection. Development, implementation, and training on safety protocols are important preventative steps (6). Laboratory personnel should be aware of immediate steps to be taken, including notification of laboratory supervisors, occupational health clinics, and local and state public health departments based on reporting regulations in their localities. These steps can reduce the risk of severe infection and possible transmission to others by direct contact. Contact tracing is not usually recommended because proper infection control techniques reduce risk to others; however, the investigations should focus on infection control, and if there is a concern about exposure to others, contact investigation should be limited to persons who might have had contact with lesion exudates (2).

What is already known on this topic?

Occupational exposures to orthopoxviruses in laboratories can result in infections. The most effective means of prevention are preexposure smallpox vaccination, training, and laboratory safety measures such as proper handling and disposal of needles. In addition, incident reporting and timeliness of seeking medical treatment for inadvertent exposures are critical components of laboratory response plans.

What is added by this report?

In November 2013, a worker in an academic laboratory inadvertently stuck his thumb with a needle being used to inoculate a mouse with wild type vaccinia virus. Despite having been vaccinated with smallpox vaccine less than one year earlier, he developed a rash on his arm and necrotic lesion on his thumb that resolved following treatment. This is the first report of a laboratory worker in the United States vaccinated against vaccinia virus according to Advisory Committee on Immunization Practices guidelines who exhibited infection after an unintentional inoculation. Recommendations to enhance worker safety were made and implemented.

What are the implications for public health practice?

Vaccination alone is insufficient as the sole preventive measure against laboratory-acquired orthopoxvirus infections. It must be complemented with effective biosafety protocols such as education of laboratory personnel, safe laboratory practice, and incident reporting.

This case report demonstrates the importance of local public health involvement with research laboratories working with organisms that might present a public health risk. Laboratory-acquired VACV infection is not nationally notifiable. However, analysis of information gathered nationally might be useful to develop and monitor best practices. It would also be useful for CDC to be aware of such occurrences to determine if improvements or changes in current recommended protocols need to be made.
